# 18 kDa Translocator Protein TSPO Is a Mediator of Astrocyte Reactivity

**DOI:** 10.1021/acsomega.3c03368

**Published:** 2023-08-15

**Authors:** Benjamin B. Tournier, Farha Bouteldja, Quentin Amossé, Alekos Nicolaides, Marcelo Duarte Azevedo, Liliane Tenenbaum, Valentina Garibotto, Kelly Ceyzériat, Philippe Millet

**Affiliations:** † Department of Psychiatry, University Hospitals of Geneva, Geneva 1206, Switzerland; ‡ Department of Psychiatry, University of Geneva, Geneva 1211, Switzerland; § Laboratory of Cellular and Molecular Neurotherapies, Center for Neuroscience Research, Clinical Neuroscience Department, Lausanne University Hospital, Lausanne 1011, Switzerland; ∥ Division of Nuclear Medicine, Diagnostic Department, University Hospitals of Geneva, Geneva 1206, Switzerland; ⊥ CIBM Center for BioMedical Imaging; NIMTLab, Faculty of Medicine, University of Geneva, Geneva 1211, Switzerland

## Abstract

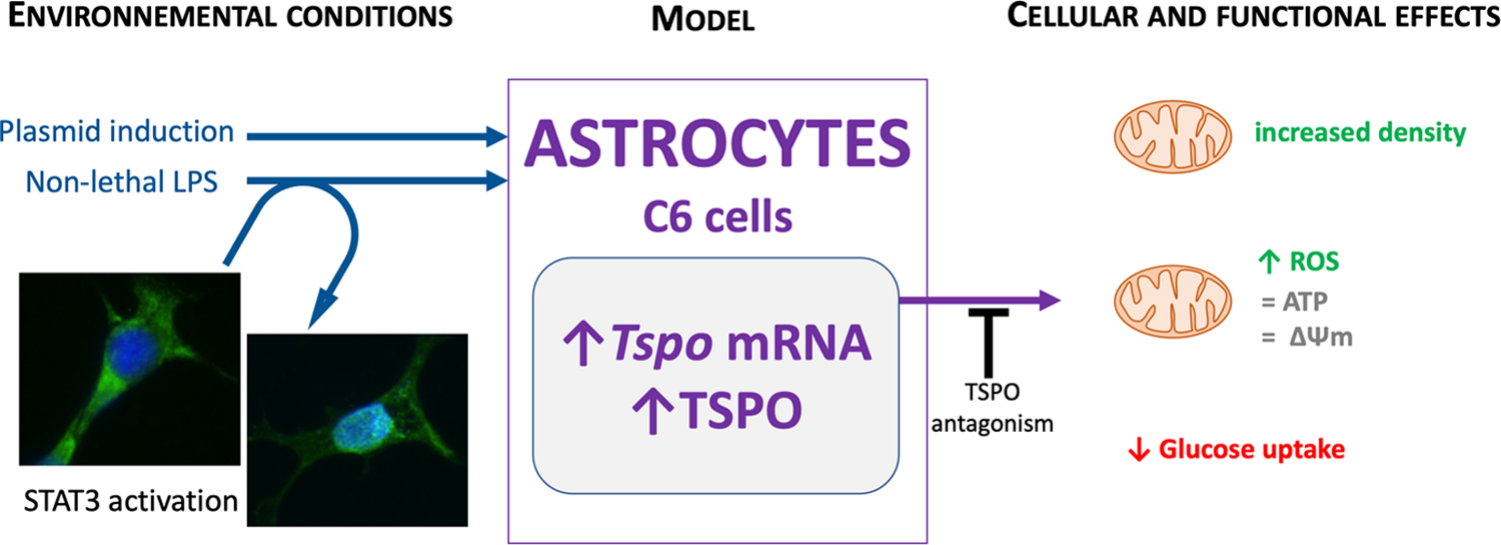

An increase in astrocyte reactivity has been described in Alzheimer’s disease and seems to be related to the presence of a pro-inflammatory environment. Reactive astrocytes show an increase in the density of the 18 kDa translocator protein (TSPO), but TSPO involvement in astrocyte functions remains poorly understood. The goal of this study was to better characterize the mechanisms leading to the increase in TSPO under inflammatory conditions and the associated consequences. For this purpose, the C6 astrocytic cell line was used in the presence of lipopolysaccharide (LPS) or TSPO overexpression mediated by the transfection of a plasmid encoding TSPO. The results show that nonlethal doses of LPS induced TSPO expression at mRNA and protein levels through a STAT3-dependent mechanism and increased the number of mitochondria per cell. LPS stimulated reactive oxygen species (ROS) production and decreased glucose consumption (quantified by the [^18^F]FDG uptake), and these effects were diminished by FEPPA, a TSPO antagonist. The transfection-mediated overexpression of TSPO induced ROS production, and this effect was blocked by FEPPA. In addition, a synergistic effect of overexpression of TSPO and LPS on ROS production was observed. These data show that the increase of TSPO in astrocytic cells is involved in the regulation of glucose metabolism and in the pro-inflammatory response. These data suggest that the overexpression of TSPO by astrocytes in Alzheimer’s disease would have rather deleterious effects by promoting the pro-inflammatory response.

## 1 Introduction

The 18 kDa translocator protein (TSPO) is a mitochondrial membrane protein implicated in many essential mitochondria-based physiological processes, including steroidogenesis, cholesterol transport, cellular bioenergetics, mitochondrial respiration, and apoptosis.^1,2^ In the brain, TSPO is used as an inflammation marker as it is upregulated in pathological conditions such as Alzheimer’s disease or amyotrophic lateral sclerosis.^3,4^ Previous studies have shown that TSPO ligands showed a decrease in lipopolysaccharide (LPS)-induced inflammation^5,6^ and a decrease in pathological marks and cell death in a mouse model of Parkinson’s disease.^7^ The TSPO knockout in a mouse model of Alzheimer’s disease induced a decrease in astrocyte reactivity and in pathological markers.^8^ These observations suggest the use of TSPO at a therapeutic level. However, in the central nervous system, even if microglia and astrocytes are now recognized as the main source of TSPO,^9,10^ its role in glial cells is largely unexplored. In addition, the cell source of TSPO overexpression depends on the pathology and the phase of the disease. In fact, in an experimental autoimmune encephalomyelitis model of multiple sclerosis, the dynamics of TSPO overexpression indicated an involvement of microglia and then of astrocytes, during the remyelination phase.^10^ In Alzheimer’s disease, the overexpression of TSPO in astrocytes occurs before the one in microglia.^11^ Thus, understanding the role of TSPO according to its cellular origin would allow better targeting of TSPO-based therapies.

Microglia have long been viewed as the sole source of TSPO and imaging studies attributed variations in TSPO expression to microglia. In mouse microglial cells, TSPO knockout reduced glycolysis, ATP production, and mitochondrial membrane potential,^12^ suggesting a role of microglial TSPO in glucose metabolism and cell energy. LPS induced TSPO overexpression in microglia, and the inhibition of TSPO leads to a decrease in the LPS-induced inflammatory reaction,^12,13^ that suggests a pro-inflammatory role of microglial TSPO. In contrast to rodents, the increase in microglial TSPO signal in human microglia may better reflect the cell proliferation than an increase in TSPO protein per cell, as observed in the cortex of Alzheimer’s disease subjects.^11,14,15^ It can be hypothesized that increases in TSPO are partly due to STAT3 since an increase in STAT3 has been described in AD and stimulation of TSPO by STAT3 has been observed in nonbrain cells.^16,17^

In contrast, no astrocytic cell proliferation accompanied the TSPO overexpression in the cortex of subjects with Alzheimer’s disease,^11,15^ which could suggest different roles of TSPO depending on the brain region as well as the cell type. Astrocytes are involved in maintaining the homeostasis of the brain parenchyma. However, under many conditions, they become reactive. This state reflects a change in function accompanied by transcriptomic and morphological alterations. This response, which can be protective or harmful for the surrounding tissue, depends on the nature and duration of the stimulation.− In Alzheimer’s disease, astrocytes participate in the elimination of extracellular debris by phagocytosing the amyloid,^22^ which can be modeled *in vitro* in cell culture.^23^ In rat C6 astrocyte cultures, it was shown that nanomolar doses of TSPO antagonists stimulated cell proliferation while micromolar doses induced cell death by apoptosis.^24^ In glioma, the TSPO knockout induced an increase in fragmented mitochondria and stimulated a glucose uptake.^25^ However, the roles of the astrocytic TSPO in the reactivity of astrocytes are not yet well understood. As an example, the role of TSPO in LPS-induced reactive oxygen species (ROS) release by astrocytes is still unresolved.

In contrast to brain cells, it was previously shown in steroid hormone-producing cells that the TSPO expression is under the control of signal transducers and activators of transcription 3 (STAT3) and mitogen-activated protein kinase (MEK/ERK) pathways. As astrocytes are shown as one of the central pivots of different pathologies, including Alzheimer’s disease,^20,21^ a better understanding of intracellular mechanisms in reactive astrocytes could be useful for the development of therapeutic strategies. Thus, we sought to clarify the role of TSPO in astrocytes during an acute exposure to a pro-inflammatory environment. We investigated the role of TSPO in the cellular mechanistic response mediated by LPS and TSPO overexpression in the C6 astrocytic cell line and the implication of TSPO in their ability to phagocytose amyloid.

## 2 Materials and Methods

### 2.1 C6 Cell Cultures

The astrocytic C6 cell line was cultured in a T75 flask in a complete medium, including RPMI 1640 (Thermo Fisher, 31870025) with 10% FBS (Thermo Fisher, 16000044), 3% penicillin/streptomycin (Thermo Fisher, 15070063), 2 mM l-glutamine (Thermo Fisher, 25030024), 1% sodium pyruvate (Thermo Fisher, 11360039), and 2% HEPES (Thermo Fisher, 15630056) at 37 °C, 5% CO2. The cells were subcultured after reaching 70–80% confluence and seeded into 6- to 96-well tissue culture plates depending on the experiments.

### 2.2 Radioligand Binding Assay

The radioligand binding assay was performed using [^125^I]CLINDE as the radioactive TSPO ligand for evaluation of the density in TSPO. [^125^I]CLINDE synthesis protocol can be found here,^26^ but briefly, the CLINDE tributyltin precursor was incubated in acetic acid with Na^125^I (PerkinElmer) and peracetic acid before purification using a reversed-phase column.

C6 cells were seeded at 1.0 × 10^5^ cells/well density in 24-well plates. After 8 h of incubation at 37 °C, 5% CO_2_ medium was replaced with fresh medium containing [^125^I]CLINDE (at 10 different concentrations from 0.005 to 1 μCi/well). The medium was supplemented with LPS from *Escherichia coli* (10 μg/mL, Sigma-Aldrich, L2630) for the treated group and with FEPPA (*N*-(2-(2-fluoroethoxy)benzyl)-*N*-(4-phenoxypyridin-3-yl)acetamide, 10 μM, ABX advanced biochemical compounds, Germany) for the determination of the nonspecific binding. After overnight incubation at 37 °C, 5% CO_2_ cells were washed three times with 50 mM Tris HCl and 50 mM MgCl_2_ buffer. The cells were detached using 300 μL/well of triple detergent buffer (Tris 1 M pH 8, NaCl, azide sodium 10%, SDS20%, NP40 (IGEPAL CA-630), deoxycholate sodium (D6750-10G), supplemented with inhibitors of proteases and phosphatases) and immediately counted using a γ counter (Wizard 3, PerkinElmer).

### 2.3 qPCR

C6 cells were seeded at a 1 × 10^5^ cells/mL density in T25 flasks. After 24 h of incubation at 37 °C, 5% CO_2_ medium was replaced with fresh medium supplemented with LPS (10 μg/mL) for the treated group. After another 24 h incubation at 37 °C, 5% CO_2_, the cells were detached using trypsin-EDTA (Thermo Fisher, 25300054) and centrifuged for 5 min at 3000*g*. Total RNA was extracted using the RNeasy mini kit (Qiagen), and cDNA synthesis was performed using the SuperScript VILO cDNA synthesis kit (Invitrogen) according to manufacturer instructions. Quantitative PCRs were performed using SYBR Green detection and PCR cycles as follows: initial denaturation 95 °C, 30 s, followed by 40 cycles of 95 °C, 15 s; 60 °C, 1 min and a final step 65 °C, 30 s and then 5 s to 95 °C (0.5 °C/sec), 15 s. The following primers were used to detect *Tspo* (fd: GCTGCCCGCTTGCTGTATCCT; rev:CCCTCGCCGACCAGAGTTATCA) and the housekeeping *Ppia* gene (fd: ATGGCAAATGCTGGACCAAA, rev:GCCTTCTTTCACCTTCCCAAA). The *Tspo* mRNA level was expressed relative to that of the *Ppia* gene expression.

### 2.4 Mitochondrial Organization

C6 cells were seeded at 1.5 × 10^5^ cells/well density in a two-chamber slide (Nunc Lab-Tek Chamber Slide System, Thermo Fisher), one chamber was used for CTL, and the other one was treated with LPS 10 μg/mL. The cells were then incubated overnight at 37 °C, 5% CO_2_. The medium was removed and replaced with preheated (37 °C) medium containing the mitochondrial staining MitoTracker solution (Thermo Fisher, M7510, a referenced method for measuring mitochondrial density^27−32^) at 150 nM and then incubated for 30 min at 37 °C, 5% CO_2_. The cells were then rinsed 2× with PBS 0.1 M and fixed using preheated (37 °C) PFA 4% for 15 min at 37 °C. After rinsing again 2× with PBS, permeabilization of the cells was done by adding a 0.1% Triton X-100/1% BSA/PBS solution for 30 s at RT before rinsing 2× with PBS. A cytoskeleton staining solution (CellMask, Thermo Fisher, A57245) was added at 1× concentration for 15 min at RT for cytoskeleton staining, and then the cells were rinsed 2× with PBS. Finally, DAPI solution was added for 10 min at RT for nucleus staining and the cells were rinsed 2× with PBS. After the last washing with PBS, the supernatant was removed, the slide was allowed to dry at RT, the boxes were removed, and a coverslip was put on top of the slide using FluorSave (Millipore). The fluorescent stains were observed with an epifluorescence Eclipse Ti2-E Nikon inverted microscope and further analyzed with ImageJ.

### 2.5 MTT Assay

To measure cell proliferation/survival, an MTT (3-(4,5-dimethylthiazolyl-2)-2,5-diphenyltetrazolium bromide) reduction assay was performed. The cells were seeded at a density of 1 × 10^5^ cells/mL in a 96-well plate. After 24 h of incubation at 37 °C, 5% CO_2_, the medium was replaced by fresh medium containing LPS at different concentrations, ranging from 50 to 830 μg/mL. After 24 h of incubation at 37 °C, 5% CO_2_, MTT (5 mg/mL) was directly added to the medium for 4 h. Medium was replaced by 100 μL of DMSO and incubated under agitation at RT for 10 min, protected from light, before the measuring of the absorbance at 570 nm.

### 2.6 Pro-caspase 3/7 Activation

The cells were plated at a density of 1.0 × 10^5^ cells/mL in a two-well chamber slides (Nunc Lab-Tek Chamber Slide System, Thermo Fisher). After 8 h of incubation at 37 °C, 5% CO_2_, the medium was replaced with fresh medium, supplemented with LPS (10 μg/mL) for the treated group. After 24 h of incubation at 37 °C, 5% CO_2_, the medium was replaced by CellEvent caspase 3 and 7 Green Detection Reagent (5 μM in 0.1 M PBS, Thermo Fisher, C10427). After 30 min of incubation at 37 °C, 5% CO_2_, images were immediately taken using the epifluorescence Eclipse Ti2-E Nikon inverted microscope. The percentage of positive cells/area (x20 magnification fields of view, around 30 cells/field) was measured as an indicator of caspase 3/7 activation and hence apoptosis.

### 2.7 MEK and STAT3 Inhibitors

C6 cells were seeded at 1.0 × 10^5^ cells/well density in 24-well plates. After 8 h of incubation at 37 °C, 5% CO_2_, MEK inhibitor (U0126, 20 μM, Sigma), STAT3 inhibitor (5,15-DPP, 20 μM, Sigma), [^125^I]CLINDE (0,5 μCi/well), and FEPPA (10 μM) were directly added to the corresponding wells. As the stock solutions of MEK and STAT3 inhibitors were diluted in DMSO, control cells were incubated with the same amount of DMSO/well. After overnight incubation at 37 °C, 5% CO_2_, the cells were washed three times with 50 mM Tris HCl, 50 mM MgCl_2_ buffer, detached using 300 μL/well of triple detergent buffer (Tris 1 M pH 8, NaCl, azide sodium 10%, SDS20%, NP40 (IGEPAL CA-630), deoxycholate sodium (D6750-10G), supplemented with inhibitors of proteases and phosphatases), and immediately counted using a γ counter. In a different set of experiments, the same procedure was reproduced with the addition of LPS (10 μg/mL) when the inhibitors were added.

### 2.8 Nuclear Translocation of STAT3

C6 cells were seeded at 1.0 × 10^5^ cells/mL in two-well chamber slides (Nunc Lab-Tek Chamber Slide System, Thermo Fisher). After 24 h of incubation at 37 °C, 5% CO_2_, the medium was replaced by medium supplemented with LPS (10 μg/mL) for treated groups and C6 medium for control. The cells were fixed with PFA 4% for 10 min at 4 °C, washed 3x in PBS 0.1 M, and then permeabilized using ice-cold 100% methanol for 15 min at −20 °C. Blocking buffer (Triton X-100 0.1%, PBS 0.1 M, BSA 5%) was then applied for 1 h following three washes with PBS 0.1 M. Then, the cells were incubated overnight at 4 °C with primary mouse anti-STAT3 antibody (1:200, Cell Signaling) diluted in Triton X-100 0.3%, PBS 0.1 M, BSA 1%. After three times washing, the cells were incubated for 1 h with secondary antibody antimouse 488 Alexa Fluor (1:500, Thermo Fisher) diluted in Triton X-100 0.3%, PBS 0.1 M, BSA 1%. The slides were then washed 3x in PBS 0.1 M and incubated at room temperature for 10 min with DAPI. Finally, the slides were washed 3x in PBS 0.1 M and a coverslip was fixed using FluorSave mounting medium.

### 2.9 ROS Production Estimation with Dihydroethidium Assay (DHE)

Cells were plated on glass coverslips at a density of 1.0 × 10^5^ cells/mL in a 12-well plate. After overnight incubation at 37 °C, 5% CO_2_, the medium was replaced by fresh C6 medium alone or supplemented with FEPPA (10 μM). After 1 h incubation, LPS (10 μg/mL) was directly added into the treated wells. After overnight incubation at 37 °C, 5% CO_2_, the glass coverslips were rapidly rinsed with 1× PBS and dropped in a 24-well plate. 300 μL of DHE (5 μM, Thermo Fisher, D23107) was added and time lapses were directly recorded with an epifluorescence Eclipse Ti2-E Nikon inverted microscope. For the first 10 min, images were acquired every 5 s, then once every 15 s for the following 10 min. Analyses were performed using the *Time Series Analyzer V3* plugin (ImageJ), and the first raw intensity of each condition was subtracted to eliminate the background. Slope values of the kinetic curves are expressed as the rate of DHE oxidation per second and representative of ROS amount.

### 2.10 ATP and Mitochondrial Membrane Potential Determination

C6 cells were seeded at 1.0 × 10^5^ cells/well density in 24-well plates for the mitochondrial membrane potential determination and at 1 × 10^4^ cells/well density in 96-well plates for the ATP quantification. After 8 h of incubation at 37 °C, the 5% CO_2_ medium was replaced with fresh medium containing LPS (10 μg/mL) or LPS and FEPPA (10 μM). After overnight incubation at 37 °C, 5% CO_2_, ATP or mitochondrial membrane potential were measured. The ATP quantification was carried out according to the recommendations of the supplier (Luminescent ATP detection assay kit, Abcam). The mitochondrial membrane potential was determined by the ratio of aggregate/monomer forms of the JC-1 dye following the manufactured protocol (JC-1 dye, Invitrogen).

### 2.11 [^18^F]FDG Uptake

C6 cells were seeded at 1 × 10^5^ cells/well density in 24-well plates. After 8 h of incubation at 37 °C, 5% CO_2_, the medium was replaced by fresh C6 medium alone or supplemented with FEPPA (10 μM) or PBR28 (10 μM) and/or LPS (10 μg/mL). After overnight incubation at 37 °C, 5% CO_2_, [^18^F]FDG (0.5 μCi/well) was added for 15 min at 37 °C, 5% CO_2_, and the cells were washed two times in PBS 0.1 M. The cells were detached using 500 μL/well of trypsin-EDTA, and the radioactivity was measured using a γ counter.

### 2.12 TSPO Vector

The human translocator protein TSPO cDNA sequence (NM_009775.4) was fused to the 14 amino acids V5 tag^33^ at the 5′ end, separated from the TSPO coding sequence by a linker sequence (CGTGATCCTCCAGTCGCGACA) and flanked by AgeI and NotI restriction sites. The DNA sequence was synthesized by GeneArt Gene synthesis (Thermo Fisher) and cloned in p*Hpa*I-EGFP AAV vector, a kind gift from McCarty and Samulski,^34^ in place of eGFP using AgeI and NotI sites. The sequence was as follows:

ACCGGTTCTAGAATGGGGAAGCCTATCCCTAACCCTCTCCTCGGTCTCGATTCTACGCGTGATCCTCCAGTCGCGACAGCCCCGCCCTGGGTGCCCGCCATGGGCTTCACGCTGGCGCCCAGCCTGGGGTGCTTCGTGGGCTCCCGCTTTGTCCACGGCGAGGGTCTCCGCTGGTACGCCGGCCTGCAGAAGCCCTCGTGGCACCCGCCCCACTGGGTGCTGGGCCCTGTCTGGGGCACGCTCTACTCAGCCATGGGGTACGGCTCCTACCTGGTCTGGAAAGAGCTGGGAGGCTTCACAGAGAAGGCTGTGGTTCCCCTGGGCCTCTACACTGGGCAGCTGGCCCTGAACTGGGCATGGCCCCCCATCTTCTTTGGTGCCCGACAAATGGGCTGGGCCTTGGTGGATCTCCTGCTGGTCAGTGGGGCGGCGGCAGCCACTACCGTGGCCTGGTACCAGGTGAGCCCGCTGGCCGCCCGCCTGCTCTACCCCTACCTGGCCTGGCTGGCCTTCACGACCACACTCAACTACTGCGTATGGCGGGACAACCATGGCTGGCGTGGGGGACGGCGGCTGCCAGAGTGAGCGGCCGC.

### 2.13 TSPO Overexpression

Cells were grown at a density of 1.0 × 10^5^ cells/well on a coverslip in 12-well plates. After 24 h of incubation at 37 °C, 5% CO_2_, the cells were transfected with the vector caring TSPO gene (125 ng/well) supplemented with sonicated salmon sperm DNA (375 ng/well). Total DNA in serum-free RPMI was mixed with polyethylenimine (PEI, 1 mg/mL, Polysciences, PEI/DNA ratio 5:1) for 15 min at RT before being added to the cell culture medium (250 μL/mL). In all experiments using the vector-induced TSPO overexpression, a plasmid caring eGFP gene (a gift from McCarty and Samulski^34^) was used as control. After 4 days, the cells underwent ROS detection analysis (as previously described). For the FEPPA (10 μM) treated group, it was directly added to the medium after 4 days of incubation, and ROS were analyzed following overnight incubation.

### 2.14 Amyloid Phagocytosis

Treated cells were transfected with the TSPO gene as described above and incubated for 48 h at 37 °C, 5% CO_2_. The cells were then detached using Trypsin-EDTA (Thermo Fisher, 25300054), centrifuged 5 min at 3000*g*, and resuspended in complete C6 medium before being seeded at 2.5 × 10^3^ cells/well density in a 96-well plate (Greiner uClear 655090) and incubated overnight at 37 °C, 5% CO_2_. The medium was then removed and replaced with phenol red-free medium containing an amyloid-β solution (HiLyte Fluor 555, AS-60480-01, AnaSpec) at 0.25 μM. Amyloid intensity was measured after 6 and 24 h using a plate reader fluorescence microscope (ImageXpress, Molecular Devices).

### 2.15 Statistical Analysis

Data are presented as individual values and mean ± SD. Two-tailed unpaired Student *t* test was performed to compare the two groups. ANOVA tests were performed with Dunnett’s (when comparing with a single control group) or Tukey’s *post hoc* test using Prism (GraphPad, San Diego, CA). The number of replicates per experiment is indicated in the figure legends.

## 3 Results

### 3.1 TSPO Is Modulated by LPS and STAT3

**Figure 1:**
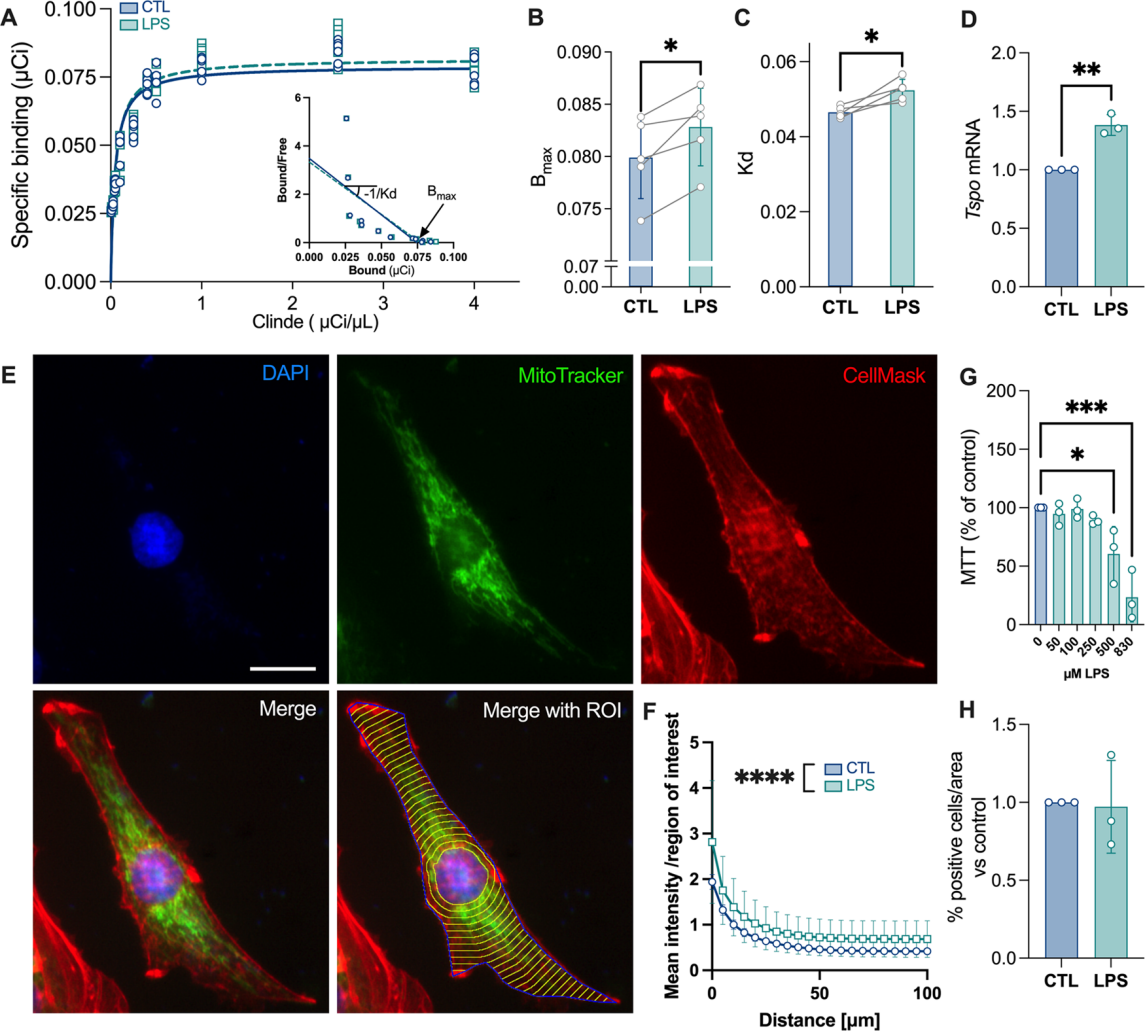
Nonlethal dose of LPS stimulates TSPO expression and density, decreases its ligand binding affinity, and increases the number of mitochondria. C6 cells were treated with LPS (10 μg/mL) and [^125^I]CLINDE (A–C) for 24 h before radioactivity measurement. (A) Mean saturation binding curve and Scatchard plot. (B, C) Indexes calculated from the Scatchard curve: total binding sites, Bmax (B), and equilibrium dissociation constant, *K*_d_ (C). Two-tailed paired Student *t* test, *p < 0.05, **p < 0.01 (*n* = 5 independent experiments with 24 replicates/group). (D) qPCR analysis of the *Tspo* gene expression. Two-tailed unpaired Student *t* test, **p < 0.01 (*n* = 3 independent experiments with 2 replicates/group). (E, F) Analysis of mitochondrial distribution in C6 cells. (E) Representative cell (untreated cell) with the nucleus (DAPI, blue), mitochondria (green, MitoTracker), cytosol (red, CellMask), the merge image, and the merge image with the regions of interest (ROIs, yellow: concentric circles starting around the nucleus; blue: cell boundary). Scale bar: 50 μm. (F) Quantification of the mitochondrial density (mean intensity per annulus) as a function of the distance to the nucleus. Two-way ANOVA, main effect of LPS (****p < 0.0001) and distance from the nucleus (****p < 0.0001) (*n* = 31–35 cells per condition). (G) Survival of cells (MTT assay) in response to 24 h LPS treatments. One-way ANOVA with the *post hoc* Dunnett’s multiple comparisons test, *p < 0.05, ***p < 0.001 (*n* = 3 independent experiments with 10–16 replicates/group). (H) % of apoptotic cells (caspase 3/7 staining). Two-tailed unpaired Student *t* test, *p* > 0.05 (*n* = 3 independent experiments with 10–11 replicates/group).

To investigate if the density of TSPO is increased in C6 astrocyte cells in response to LPS (10 μM, 24 h), and if this treatment induces an alteration of its ligand binding affinity, a saturation curve, using [^125^I]CLINDE, was carried out (Figure 1A). This allows the expression of the Scatchard plot and the calculation of the associated values. The total density in TSPO (Bmax) was increased in response to LPS (two-tailed paired Student *t* test, **p = 0.0049, Figure 1B). Conversely, the affinity (1/kd) of TSPO for its binding to [^125^I]CLINDE was decreased (two-tailed paired Student *t* test, *p = 0.040, Figure 1C). *Tspo* mRNA levels are also stimulated by LPS (two-tailed unpaired Student *t* test, **p = 0.0017, Figure 1D). In addition, the LPS treatment increased the density in mitochondria regardless of the distance from the nucleus (Figure 1E,F and Supplemental Figure 1, two-way ANOVA: main effect of LPS, *F*_1,1344_ = 152.1; ****p < 0.0001; main effect of the distance from the nucleus, *F*_20,1344_ = 58.22; ****p < 0.0001). Importantly, the LPS treatment did not alter the cell proliferation up to a dose of 250 μM (one-way ANOVA, LPS dose effect: *F*_5,12_ = 14.22, ***p = 0.0001, with the Dunnett’s *post hoc* test, Figure 1G) and did not induce apoptosis (% of cells with caspase 3/7 activation, two-tailed unpaired Student *t* test, *p* > 0.05, Figure 1H).

**Figure 2:**
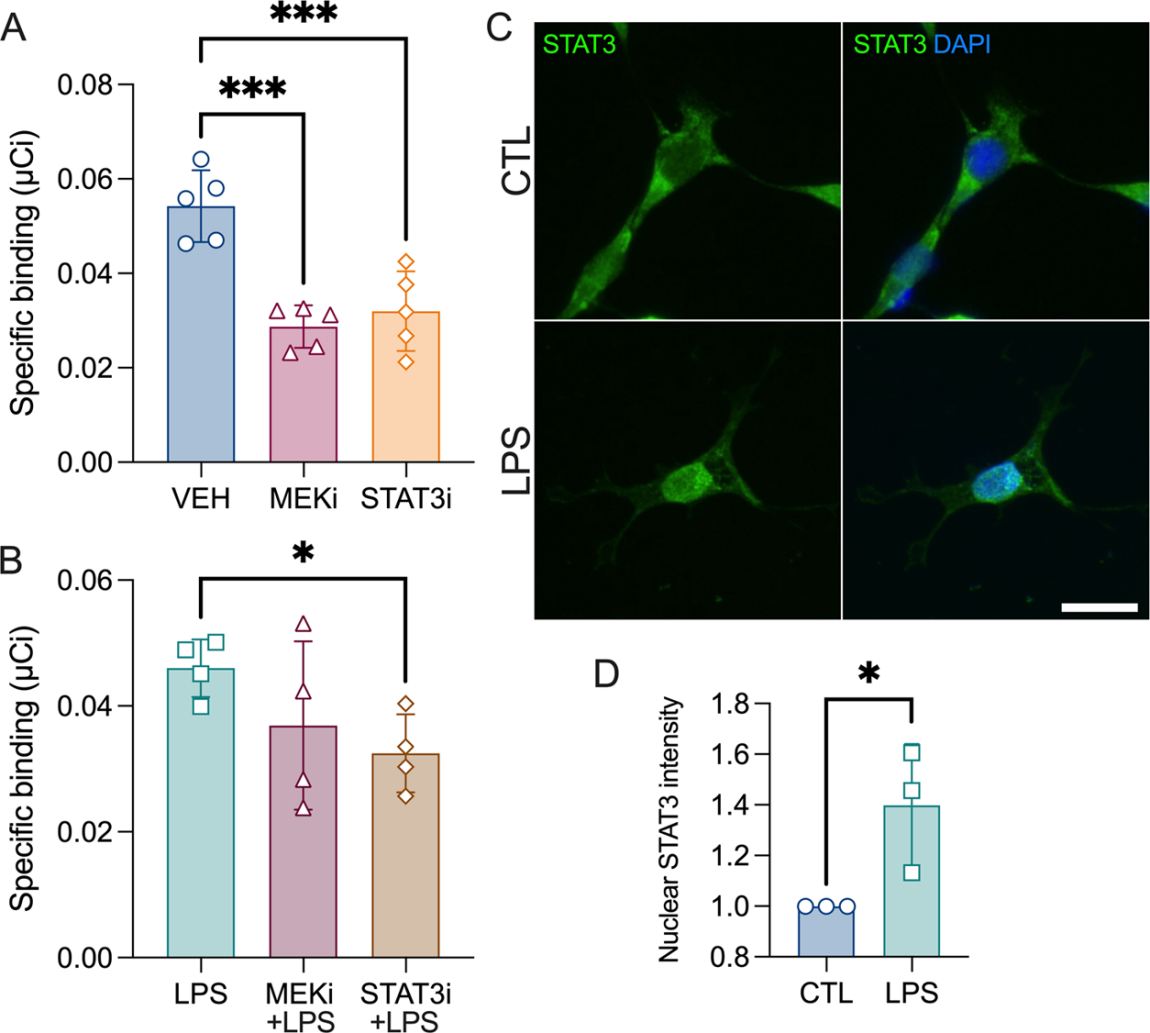
TSPO is under the control of STAT3 and ERK pathways. (A) C6 cells were treated with 5,15-DPP (STAT3 inhibitor, 20 μM) or U0126 (MEK inhibitor, 20 μM) and [^125^I]CLINDE for 24 h before radioactivity measurement for the determination of the specific binding of [^125^I]CLINDE. One-way ANOVA with the *post hoc* Dunnett’s multiple comparisons test, ***p < 0.001 (*n* = 5 independent experiments with 3 replicates/group). (B) Specific binding of [^125^I]CLINDE in C6 cells treated with LPS in response to 5,15-DPP (STAT3 inhibitor, 20 μM) or U0126 (MEK inhibitor, 20 μM). Student *t* test, *p < 0.05 (*n* = 4 independent experiments with 3 replicates/group). (C) Representative example of C6 cells stained with STAT3 (green) and DAPI (blue) in control and LPS conditions. Scale bar: 50 μm. (D) Measure of the nuclear STAT3 intensity signal at baseline and in response to LPS. Two-tailed Student *t* test, *p < 0.05 (*n* = 3 independent experiments with 20 cells analyzed/experiment).

To identify the underlying mechanisms of TSPO increase, C6 cells were first pretreated with two potential TSPO regulators. As shown in Figure 2A, inhibition of both STAT3 and ERK pathways induced a reduction in TSPO density (one-way ANOVA, treatment effect: *F*_2,12_ = 19.42, ***p = 0.0002, with the Dunnett’s *post hoc* test). Then, to determine if STAT3 and ERK pathways play a role in LPS-induced TSPO, C6 cells were cotreated with LPS and either 5,15-DPP (STAT3 inhibitor) or U0126 (MEK inhibitor). The one-way ANOVA did not reach significance (main effect of the treatment: *F*_2,9_ = 2.390, *p* = 0.14), but the two-by-two comparison showed that the blockage of STAT3, but not that of ERK, induced a reduction in TSPO density (Student *t* test, *p = 0.013, Figure 2B). Importantly, the addition of LPS induced the nuclear translocation of STAT3, demonstrating the activation of the pathway (unpaired two-tailed Student *t* test, *p = 0.046, Figure 2C,D).

### 3.2 TSPO Plays a Role in LPS-Induced ROS and LPS-Induced Reduction in [^18^F]FDG Cell Uptake

**Figure 3:**
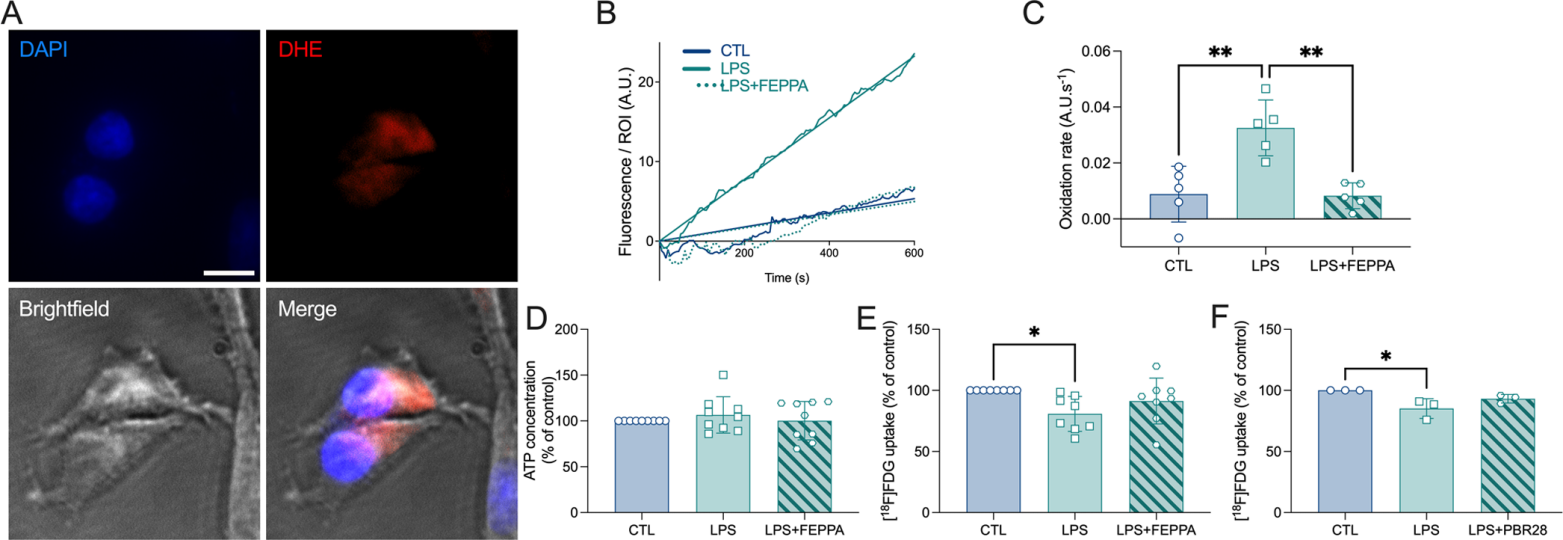
TSPO antagonist controls LPS-induced ROS production. (A) Representative example of oxidized DHE staining (red) in C6 cells using bright-field (gray) and DAPI (blue) as landmarks. Scale bar: 10 μm. (B) Representative example of time-lapse measurement of DHE loading (fluorescence per region of interest, ROI) in CTL, LPS-treated and LPS+FEPPA-cotreated C6 cells. A.U.: arbitrary unit. (C) Corresponding oxidation rates. Each individual value corresponds to one recording of 10 min. A.U.: arbitrary unit. One-way ANOVA with the *post hoc* Tukey’s multiple comparisons test, **p < 0.01 (*n* = 5 independent experiments with 1 ROI/experiment). (D) ATP production (expressed as % of control) in CTL, LPS-treated, and LPS+FEPPA-cotreated C6 cells. One-way ANOVA, *p* > 0.05 (*n* = 9 independent experiments with 3 replicates/experiment). (E) [^18^F]FDG uptake (expressed as % of control) in CTL, LPS-treated and LPS+FEPPA-cotreated C6 cells. One-way ANOVA with the *post hoc* Tukey’s multiple comparisons test, *p < 0.05 (*n* = 8 independent experiments with 3 replicates/experiment). (F) [^18^F]FDG uptake (expressed as % of control) in CTL, LPS-treated, and LPS+PBR28-cotreated C6 cells. One-way ANOVA with the *post hoc* Tukey’s multiple comparisons test, *p < 0.05 (*n* = 3 independent experiments with 3 replicates/experiment).

To further identify the role of TSPO in the cell response to LPS, we next investigated its impact on ROS production using the cytosolic superoxide indicator dihydroethidium (DHE). Although the DHE fluorescence was almost undetectable in untreated cells, it is significantly increased in cells treated with LPS (Figure 3A).

The time-lapse measurement of the DHE loading (Figure 3B) is used to calculate the slope of the curve, corresponding to the oxidation rate that reveals the production of ROS (Figure 3C). LPS induced a significant ROS overproduction that is reversed by the presence of FEPPA, a TSPO antagonist (one-way ANOVA, treatment effect: *F*_2,12_ = 13.1, ***p = 0.001, with the Tukey’s *post hoc* test).

The ATP production was not modified by LPS (Figure 3D, one-way ANOVA, treatment effect: *F*_2,24_ = 0.45, *p* > 0.05). In contrast, the [^18^F]FDG uptake was significantly decreased by LPS, and the TSPO antagonism using FEPPA partially reversed this effect (Figure 3E, one-way ANOVA, treatment effect: *F*_2,21_ = 4.01, *p = 0.033, with the Tukey’s *post hoc* test indicating a significant difference between control- and LPS-treated cells, *p* = 0.026, and the absence of difference between LPS+FEPPA and either control- or LPS-treated cells, *p* > 0.05). The same observation was made using PBR28 instead of FEPPA as a TSPO antagonist (Figure 3F, one-way ANOVA, treatment effect: *F*_2,6_ = 6.46, *p = 0.031, the Tukey’s *post hoc* test indicated a significant difference between control- and LPS-treated cells, *p* = 0.027, and the absence of difference between LPS+FEPPA and either control- or LPS-treated cells, *p* > 0.05).

### 3.3 TSPO Overexpression Induces ROS Production

As the blockage of TSPO reduced the LPS-induced ROS production, we speculated that TSPO may be directly associated with ROS production. To verify our hypothesis, we transfected C6 cells with a human TSPO plasmid overexpressing TSPO (independent of the presence of LPS).

**Figure 4:**
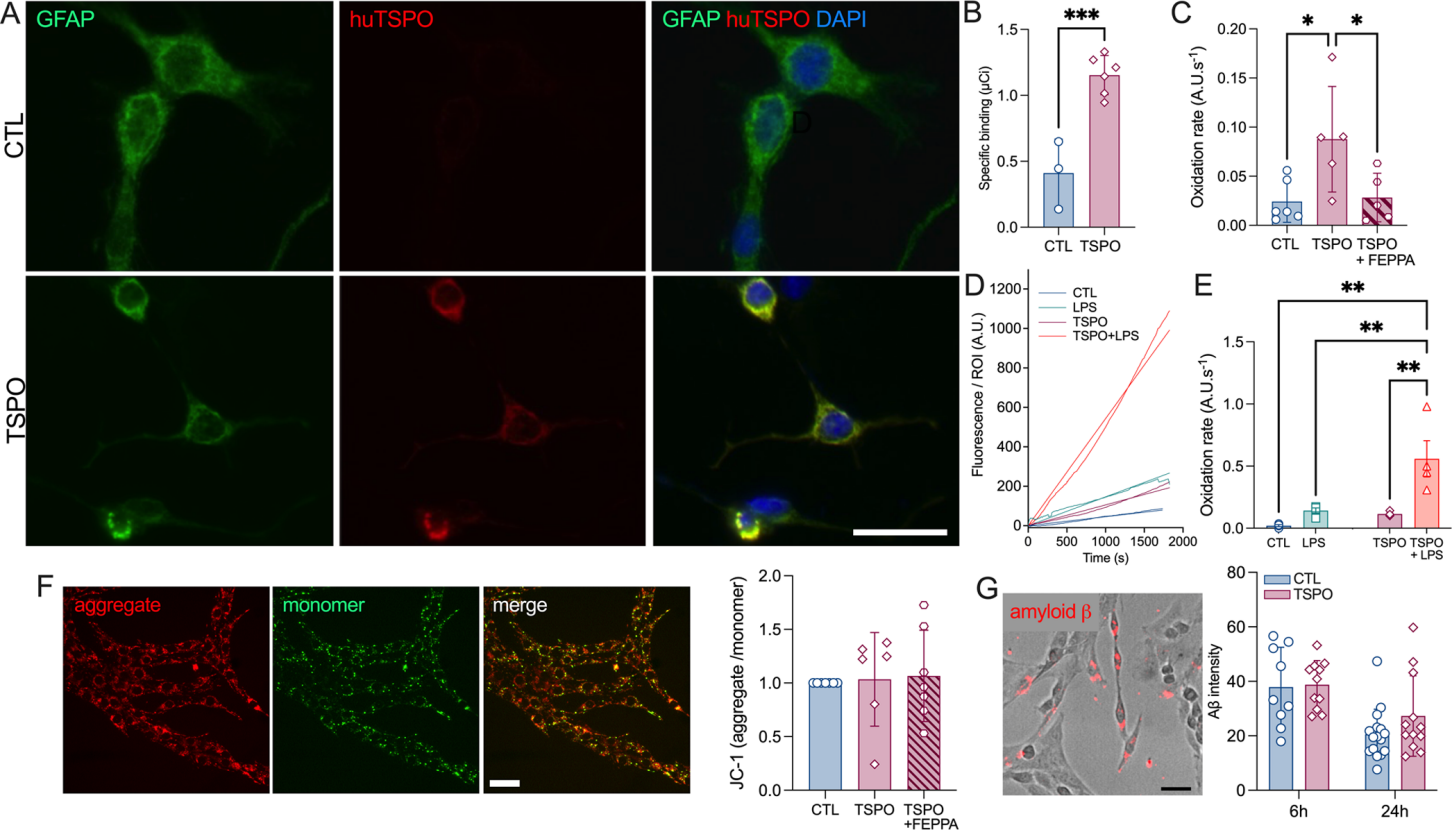
TSPO overexpression stimulates baseline and LPS-induced ROS production. (A) Representative example of human TSPO positive cells in response to a human TSPO plasmid transfection assay. Scale bar = 50 μm. (B) Specific binding of [^125^I]CLINDE in C6 cells treated with the human TSPO plasmid. Two-tailed unpaired Student *t* test, ***p < 0.001 (*n* = 3–6 independent experiments with 3 replicates/experiment). (C) Oxidation rates in C6, C6-TSPO overexpressing cells, and C6-TSPO overexpressing cells treated with FEPPA (10 μM). One-way ANOVA with the *post hoc* Tukey’s multiple comparisons test, **p < 0.01 (*n* = 5–6 independent experiments with 2 replicates/experiment). (D, E) Time-lapse and corresponding oxidation rates in C6 and C6-TSPO overexpressing cells at baseline and in response to LPS (10 μg/mL). Two-way ANOVA with the *post hoc* Tukey’s multiple comparisons test, *p < 0.05, **p < 0.01 (*n* = 4 independent experiments with 1 ROI/experiment). (F) Mitochondrial membrane potential as assessed by the JC-1 staining (aggregate/monomer ratio). Scale bar = 100 μm. One-way ANOVA, *p* > 0.05. (G) Representative example of the amyloid-β phagocytosis and mean intensity of intracellular amyloid-β at 6 and 24 h following the addition of amyloid-β in cell media. Two-way ANOVA, *p* > 0.05 (*n* = 9–18 cells per condition). Scale bar: 100 μm.

Immunofluorescence experiment confirmed the presence of human TSPO in transfected cells as well as the significant increase in [^125^I]CLINDE binding (unpaired two-tailed Student *t* test, ***p = 0.0008, Figure 4A,B). The ROS production is increased in C6 cells overexpressing TSPO as compared to the control group, and this effect was fully reversed by the TSPO antagonist FEPPA (one-way ANOVA, treatment effect: *F*_2,13_ = 5.24, *p = 0.021, with the Tukey’s *post hoc* test, Figure 4C). Then, to determine the impact of TSPO overexpression on LPS-induced ROS production, a new set of experiments was performed in C6 cells overexpressing TSPO at baseline and in response to LPS (Figure 4D). LPS and TSPO overexpression in C6 cells produced a significantly higher rate of ROS than untreated cells when a two-by-two comparison was used (unpaired two-tailed Student *t* test, **p = 0.0015 and ***p = 0.0003), confirming our previous observation. However, when ANOVA was used, ROS was highly increased in LPS-treated TSPO transfected cells compared to the other groups (i.e., control, LPS, and TSPO transfected cells, two-way ANOVA, TSPO overexpression effect: *F*_1,12_ = 11.9, **p = 0.0048; LPS effect: *F*_1,12_ = 14.74, **p = 0.0024; interaction effect: *F*_1,12_ = 4.77, *p = 0.0496, with the Tukey’s *post hoc* test, Figure 4E).

### 3.4 TSPO Overexpression Did Not Change the Mitochondrial Membrane Potential or the Phagocytosis Capacity

In response to the TSPO overexpression, we did not observe alterations in the mitochondrial membrane potential and cell phagocytosis ability. In fact, the mitochondrial membrane potential (aggregated JC-1/monomeric JC-1 signal) was unmodified (Figure 4F, one-way ANOVA, *F*_3,23_ = 0.078, *p* = 0.97) as well as the phagocytosis of amyloid peptides (Figure 4G, two-way ANOVA, TSPO overexpression effect: *F*_1,44_ = 1.18, *p* = 0.28; time effect: *F*_1,44_ = 17.17, *p* = 0.0002; interaction effect: *F*_1,44_ = 0.7, *p* = 0.40).

## 4 Discussion

The study presented here aimed to highlight the role of astrocytic TSPO in acute LPS-mediated inflammatory response. Our study shows that TSPO is overexpressed in response to LPS by a STAT3-dependent mechanism and that its function is to participate in the control of ROS production by astrocytes at baseline and in response to LPS. Overall, these data show a facilitating role of TSPO in the pro-inflammatory response.

The realization of the Scatchard effect on living cells made it possible to show two effects of LPS: an overproduction of TSPO and a reduction in the affinity of TSPO for its ligand [^125^I]CLINDE. The reduced affinity could originate from an adaptation of the complexity of the TSPO polymers. Indeed, in connection with the surrounding environment, the multimerization of the TSPO evolves, in a dynamic way.^24,35−37^ However, as the activity of TSPO seems to be dependent on its multimeric state, a modification of the affinity could suggest a modification of the dimerization states and therefore of the functional efficiency. Thus, the presence of LPS could induce an alteration of the TSPO polymers, leading to a modification of TSPO-related transmission. However, further studies are still needed to confirm this idea. The increase in TSPO density is at least partly related to the increase in transcription of the *Tspo* gene, as shown by the upregulation of mRNA levels. This stimulation appears under the control of nuclear translocation of STAT3, a transcription factor whose target sequence is present in the *Tspo* promoter.^17^ This synthetic stimulation corroborates previous studies carried out on microglial cell cultures^38,39^ and non-brain-type cells.^17,40,41^ Conversely, the ERK pathway which participates in the basal expression of TSPO^17^ is not involved in the response to LPS, at least under our stimulation conditions. The ERK pathway was involved in the upregulation of TSPO in response to Parkinson’s disease-linked neurotoxins in SH-SY5Y cells,^42^ thus indicating the multiple pathways of TSPO activation and/or the cell-type effects in the cellular response to external aggressions. LPS also induces an increase in the number of mitochondria without having any impact on their spatial organization within cells or on cell proliferation. This observation corroborates the mitochondrial fission observed in myotubes, bone-marrow-derived macrophages, and monocyte-derived macrophages in response to LPS.^43,44^ The increase in the number of mitochondria could mechanically induce an increase in the number of TSPO proteins. However, the presence of a stimulation of the nuclear translocation of STAT3 suggests that the increase in the density in TSPO is not only the reflection of mitochondrial fission but the consequence of both an increase in the number of TSPO sites by mitochondria and the number of mitochondria. These data corroborate those obtained in the TgF344-AD rat model, a model of Alzheimer’s disease, in which a TSPO increase was shown in the astrocyte population with an increase in the number of targets per cell, in the absence of cell proliferation.^11^

The simultaneous effect of increasing the TSPO density and decreasing its affinity for its ligand could also have consequences on the interpretation of *in vivo* imaging. The analysis of TSPO images by PET or SPECT is essentially carried out by measuring the binding potential (BP), which reflects the receptor density. An increase in BP is mainly interpreted as an increase in *B*_max_, due to the following formula: BP = *B*_max_/*K*_d_. However, we showed herein that not only *B*_max_ but also *K*_d_ increases. If this is the case in certain pathologies, an underestimation of the real increase in the density of the TSPO could be made when considering BP. A previous study observing the variation in ligand binding (i.e., an alteration in BP) without alteration in the quantity of TSPO proteins (as measured by Western blot) supports the idea of variation in the binding capacity of ligands depending on the state of multimerization of the TSPO.^45^ In this same idea, treatment by irradiation of proteoliposomes made it possible to show that the polymerization of TSPO induces an increase in the binding of PK11195, another TSPO antagonist.

One of the functional consequences of TSPO modifications in the cellular response to LPS is an increase of mitochondrial ROS production. This observation makes it possible to clarify one of the roles of the astrocytic TSPO, as a mediator of the response to an inflammatory environment. Our observations also showed that the increase in ROS is blocked by the pharmacological inhibition of TSPO, confirming the studies in mammary carcinoma and cardiomyocyte cell lines using genetic inhibition of TSPO.^40,46^ This ROS stimulation effect is also obtained by the overexpression of TSPO, apart from the presence of pro-inflammatory stimulation. In addition, the presence of a high density of TSPO has a synergistic effect on the production of ROS in response to LPS. These observations demonstrate the active role of TSPO in ROS production, and its extent in the cellular response to LPS. The LPS-induced ROS production in astrocyte had already been observed^47−50^ as LPS-induced astrocyte alterations *in vivo*.^51^ However, astrocytes do not appear to produce ROS in response to LPS.^52^ It is possible that the single-injection protocol of LPS, the dose, or the timing of ROS measurement failed to confirm what was observed in culture. Indeed, numerous peripheral LPS injection protocols have produced a variety of neuroinflammation results.^53^ Further studies are needed to clarify this point.

This facilitating effect of TSPO in increasing the inflammatory response that we demonstrated is corroborated *in vivo* by the presence of a decrease in the ability of LPS to induce inflammation in animals previously treated with a TSPO antagonist.^5,6^ Microglia also shows an ameliorating effect of pharmacological TSPO inhibition upon LPS exposure.^54^ Such a protective effect of TSPO inhibition has also been hypothesized in various other pathologies (multiple sclerosis, Alzheimer’s disease, and Parkinson’s disease^7,8,55^) and could therefore indicate a pro-inflammatory function of the TSPO.

The increase in TSPO by LPS did not induce a variation in ATP production, although the induction of TSPO in a naturally lacking cell model stimulates the production of ATP^56^ and the inhibition of TSPO decreases ATP.^12^ This observation could reflect a dose- or cell-type effect, as the exogenous increase in TSPO was performed in the Jurkat cell line^56^ and the TSPO inhibition in BV2 cells.^12^ The glucose uptake, another marker of astrocyte function, is decreased in the presence of LPS, which corroborates observations made previously on nonbrain cells.^57,58^ In the brain, LPS can increase or decrease glucose uptake, depending on the dose.^59,60^ Moreover, we showed that the decrease in glucose consumption is partly reversed by TSPO inhibition using FEPPA, suggesting a role for TSPO in the control of astrocyte function. This idea is reinforced using another TSPO antagonist, PBR28, which leads to the same conclusion: the involvement of TSPO in LPS-induced glucose uptake depletion. As bimodal effects of TSPO ligands have been observed in terms of cell survival,^24,45^ it will be interesting to highlight whether different dosages of LPS can lead to TSPO transmission-induced opposite effects. Thus, our data suggest that LPS induced TSPO overexpression, which led to a decrease in glucose uptake. This hypothesis is supported by a previous study showing that the TSPO knockout leads to an increase in glucose consumption.^25^ Supporting the idea of a role for TSPO in glucose metabolism, PK11195 was shown to regulate glucose pathways in mice and zebrafish.^61,62^ It could therefore be hypothesized that the increase in TPSO is related to hypometabolism observed in AD patients.

To further analyze whether TSPO also plays a role in the phagocytosis capability of astrocytes, we measured the amyloid β internalization. We showed that the overexpression of TSPO has no effect on the ability of astrocytes to phagocytose amyloid. Thus, even if in Alzheimer’s disease brain, astrocytes are reactive, overexpressed TSPO and performed amyloid phagocytosis to eliminate amyloid,^63^ our data suggest that TSPO does not seem to control such function. Interestingly, TSPO is required for the induction of apoptosis because of glutamate exposure,^64^ and our data demonstrated that TSPO is required for the LPS-induced ROS production and LPS-induced decrease in glucose uptake. Taken together, these observations tend to prove a global mechanism of TSPO on cellular metabolism in response to pro-inflammatory exposure. TSPO could appear as a mediator of astrocyte function, granting a pro- or anti-inflammatory profile and regulating cellular activity until inducing apoptosis.

Interestingly, the presence of LPS, an increase in the activated form of STAT3, an increase in TSPO density, and an increase in ROS production were described in AD brain.^16,65−71^ More importantly, the inhibition of STAT3 by systemic treatment in an AD mouse model induced a reduction in inflammation and accumulation of pathological markers.^68^ By a cell-type-specific approach, it has been shown that inhibition of STAT3 in the astrocytes induced the same effect, thus clarifying the role of astrocytic STAT3.^72^ In this same idea, the knockout of TSPO in an AD mouse model induces a decrease in astrocytosis and accumulation of abnormal forms of Tau and amyloid.^8^ Thus, the environmental deleterious conditions in AD with LPS and Aβ among others may represent the origin of the TSPO upregulation via STAT3. This body of evidence suggests a pro-inflammatory role of the TSPO from astrocytes.

Some limitations must be made. In this study, only cultures of the C6 immortal cell line were used. These cells do not display all of the phenotypic and genetic characteristics of astrocytes,^73^ which is a limitation to the interpretation of the results. Interpretation of the results obtained must therefore take this bias into account, and further in situ studies will be required to confirm the roles of TSPO. It remains possible that the effects we observed are partly due to a modification of the cellular environment induced by LPS (i.e., modification of cytokine production, etc.) which would intervene in combination with TSPO. However, as the use of FEPPA induced a return to normal in most of our measurements, these interaction effects (which we cannot formally exclude) appear to be negligible. Phagocytosis was measured with TPSO overexpression to demonstrate its direct role in this process. Future analyses under various conditions of astrocyte reactivity (stimulation with LPS) will be required to validate this observation. In the present report, we have classified FEPPA as an antagonist, as it blocked the responses induced by either LPS or TSPO overexpression. However, it is known that the simple antagonist/agonist definition is insufficient to understand the effects of TSPO ligands especially since these functions can be modulated by different factors.^37,74^

## Conclusions

All our observations converge to conclude that LPS induces an upregulation of TSPO density in addition to a change in its functions, which would lead to an overproduction of ROS and a reorganization of cellular functions. Our study therefore suggests that TSPO from astrocytes plays roles in glucose metabolism and pro-inflammatory pathways. As ROS production is a well-known inducer of pro-inflammatory cytokines,^75−78^ it may be suggested that TSPO-induced ROS would have rather deleterious effects in AD.

## Supporting Information Available

Example of MitoTracker staining in control and LPS-treated cells (PDF)
