# Supplementary material for: 18 kDa Translocator Protein TSPO Is a Mediator of Astrocyte Reactivity
Source: ACS Omega. 2023 Aug 15;8(34):31225–36. doi: 10.1021/acsomega.3c03368 (PMC10468775; doi:10.1021/acsomega.3c03368)
Supplement: Supplementary file 1 — ao3c03368_si_001.pdf [file ao3c03368_si_001.pdf]

The 18kDa translocator protein TSPO is a mediator of astrocyte reactivity.

Benjamin B. Tournier[a,b], Farha Bouteldja[b], Quentin Amossé[b], Alekos Nicolaides[b], Marcelo Duarte Azevedo[c], Liliane Tenenbaum[c], Valentina Garibotto[d], Kelly Ceyzériat[a,b,d], Philippe Millet[a,b,*]

[a]Department of Psychiatry, University Hospitals of Geneva, Switzerland
[b]Department of Psychiatry, University of Geneva, Switzerland
[c]Laboratory of Cellular and Molecular Neurotherapies, Center for Neuroscience Research, Clinical Neuroscience Department, Lausanne University Hospital, Switzerland
[d]Division of Nuclear medicine, Diagnostic Department, University Hospitals of Geneva, Switzerland; CIBM Center for BioMedical Imaging; NIMTLab, Faculty of Medicine, University of Geneva, Geneva, Switzerland

*Correspondence:
Prof Philippe Millet, PhD
University Hospitals of Geneva
Department of Psychiatry
Avenue de la Roseraie, 64
1205 Geneva, Switzerland
Tel: +41 79 553 6376
E-mail: philippe.millet@hcuge.ch

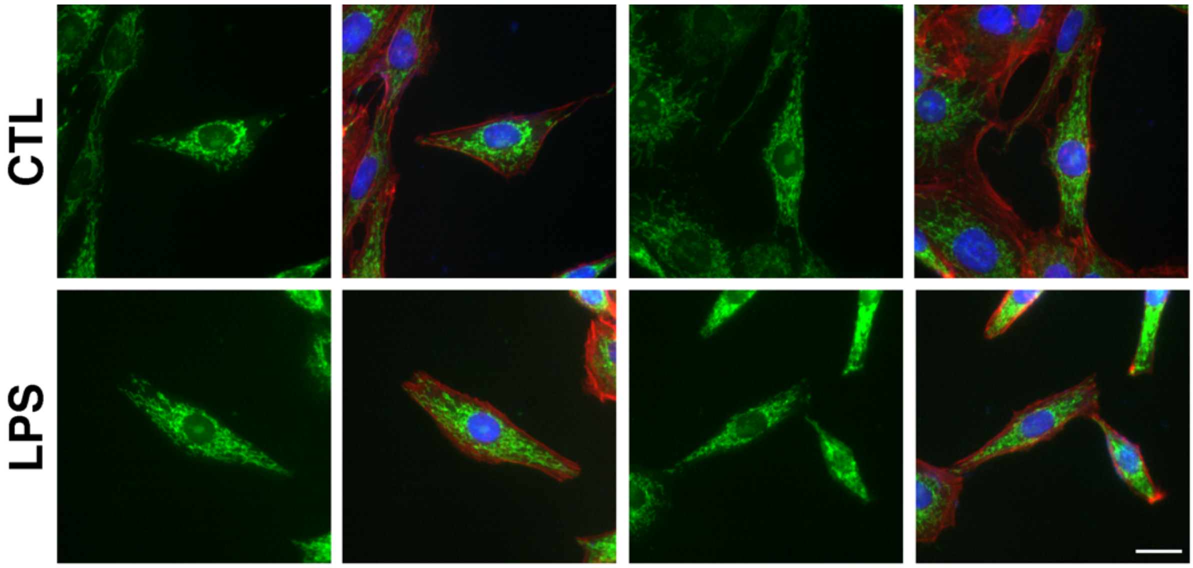

Supplemental Figure 1. Example of MitoTracker staining.

Representative cells with the MitoTracker staining (green, mitochondria) and the merge with the nucleus (DAPI, blue) and the cytosol (red, CellMask). Upper line: untreated cells; lower line: LPS-treated cells. Scale bar: 50 µm.
